# Surgical Site Infections at Shirati KMT Hospital in Northeastern Tanzania

**DOI:** 10.7759/cureus.34573

**Published:** 2023-02-02

**Authors:** Dulguun Bayardorj, Pichaya Promsatit, Bwire M Chirangi, Eiman Mahmoud

**Affiliations:** 1 Department of Global Health, College of Osteopathic Medicine, Touro University California, Vallejo, USA; 2 Hospital Administration, Shirati KMT Hospital, Shirati, TZA

**Keywords:** global surgery, wound infection, post surgery complication, global health, hospital acquired infection, surgical site infection

## Abstract

Despite improved guidelines for surgical practices and better surgical methods and tools, surgical site infection (SSI) is still a common cause of morbidity and mortality with increased rates in resource-limited nations. In Tanzania, there is limited data on SSI and associated risk factors for developing an effective surveillance system for SSI. In this study, we aimed to establish for the first time the baseline SSI rate and its associated factors at the Shirati KMT Hospital in Northeastern Tanzania. We collected hospital records of 423 patients who had undergone major and minor surgeries between January 1 and June 9, 2019, at the hospital. After accounting for incomplete records and missing information, we analyzed a total of 128 patients and found an SSI rate of 10.9% and performed univariate and multivariate logistic regression analyses for elucidating the relationship between risk factors and SSI. All patients with SSI had undergone major operations. Moreover, we observed trends of increased association of SSI with patients who are 40 or younger, female, and had received antimicrobial prophylaxis or more than one type of antibiotics. In addition, patients who had received an American Society of Anesthesiologists (ASA) score of II or III, as one category, or undergone elective operations or operations lasting longer than 30 minutes were prone to develop SSI. Although these findings were not statistically significant, both univariate and multivariate logistic regression analyses showed a significant correlation between clean contaminated wound class and SSI, consistent with previous reports. The study is the first to elucidate the rate of SSI and its correlated risk factors at the Shirati KMT Hospital. We conclude that, based on the obtained data, clean contaminated wound class is a significant predictor of SSI at the hospital and that an effective surveillance system for SSI should begin with adequate record keeping of all patients’ hospitalization and an efficient follow-up system. Moreover, a future study should aim to explore more widespread SSI predictors such as premorbid illness, HIV status, duration of hospitalization prior to operation, and type of surgery.

## Introduction

Surgical site infection (SSI) is defined as a wound infection occurring within 30 days of operation and is one of the most common types of healthcare-associated infection worldwide, accounting for 10 to 25% of all hospital-acquired infections [1,2]. SSI is associated with an increased financial burden, longer hospitalizations, and higher morbidity and mortality [1,3,4]. In the United States, it is estimated that 2-4% of all inpatient surgeries result in SSIs and that each SSI can cost about $90,000 [5,6]. Low- and middle-income countries have been reported to have similar costs but higher financial burdens due to their economic status [7]. Despite the employment of improved guidelines for pre- and post-operation practices, better sterilization methods, and upgraded surgical tools, SSI-associated morbidity and mortality are widely observed even in basic life-saving operations including cesarean sections and appendectomies in low-income countries [8]. The rate of SSI has been reported to be at the higher end of the global range of 2.5 to 30.9% in regions such as sub-Saharan Africa [9-13]. In Tanzania, SSI remains to be one of the main causes of morbidity and mortality, with the rate ranging from 10% to 26% [14-16]. Previous studies at tertiary and district hospitals in Tanzania reported SSI rates of 19.4% and 24%, respectively [14,15]. The most recent study on SSI in Tanzania was at Buganda Medical Centre in Northwestern Tanzania which reported an SSI rate of 26% [16]. However, the current data on SSI and risk factors of SSI across hospitals in Tanzania is significantly limited and outdated. Surveillance of SSI along with feedback of evidence-based information to surgeons has been reported to be an important part of strategies to reduce SSI risk [17]. Therefore, it is important to elucidate the SSI rate and generate sufficient data on risk factors associated with SSI to design an effective surveillance system for SSI. Our study is the first to establish the baseline SSI rate and explore predictive factors at Shirati KMT Hospital in Northeastern Tanzania.

## Materials and methods

This cross-sectional retrospective study involved the collection of handwritten patients’ records and clinical notes of both major and minor surgeries at Shirati KMT Hospital, Tanzania between January 1 and June 9, 2019. The study was approved with an exempt status by the Touro University California Institutional Review Board (IRB) M-1391. The handwritten data, which did not contain patients’ names, was transcribed into an Excel sheet for further statistical analysis. A total of 423 patients’ demographic and clinical information was obtained. After patients with missing records were excluded from analysis, a total of 128 patients’ data was used to determine the relationship between SSI and each of the following predictors: demographic (age and gender) and pre- and intra-operative factors (antimicrobial prophylaxis, American Society of Anesthesiologists (ASA) scoring, operation types and length, wound class, and anesthesia types). SSI rate was defined as the percentage of patients with SSI that were exposed to a predictor. The data was further analyzed using 2x2 contingency tables, and the Chi-square test was used for determining the significance of the correlation between SSI and each predictor. Additionally, multivariate logistic regression analysis was performed to determine SSI prediction based on antimicrobial prophylaxis, major and elective operations, and clean contaminated wound class. Statistical significance was defined as a p-value of less than 0.05.

## Results

Demographic characteristics associated with SSI

Overall, there were more females in the study than males: 99 vs 29 (Table 1). SSI was found in 14 patients out of the total 128, giving an overall SSI rate of 10.9%. The SSI rate in females was 11.1% while males had an SSI rate of 10.3% (Table 2). The mean age associated with SSI was 28.6 years, which was younger compared to the group that did not develop SSI; 30.7 years. This, however, was not statistically significant at p-value=0.34 (Table 1). SSI rates for those older than 40 and those 40 or younger were 7.7% and 11.8%, respectively (Table 2). SSI was not significantly associated with either gender or the two different age groups.

**Table 1:** Baseline characteristics between patients with and without Surgical Site Infection N: number of patients; std: standard deviation; yr: year; * p-value=0.34; ASA: American Society of Anesthesiologists; Abx: antibiotic prophylaxis; + p-value=0.26

Independent Factor		Yes SSI (N=14)	No SSI (N=114)
Female		11	88
	%	78.6	77.2
Age (yr)*		28.6	30.7
	std	17.4	17.3
Major Operation		14	99
	%	100	86.8
Emergency		6	64
	%	42.9	56.1
Surgery Duration+		42.1	38.9
	std	20.3	17.4
ASA Score I		4	39
	%	28.6	34.2
ASA Score II		10	74
	%	71.4	64.9
ASA Score III		0	1
	%	0.0	0.9
Clean wound		12	111
	%	85.7	97.4
Spinal Anesthesia		7	64
	%	50	56.1
General Anesthesia		6	44
	%	42.9	38.6
Abx use		13	99
	%	92.9	86.8
2 > Abx		8	49
	%	57.1	43.0

**Table 2:** Association of demographic factors with Surgical Site Infection at Shirati KMT Hospital n: number of patients in percentage; N: number of patients with or without SSI; M: male; F: female; CI: confidence interval

Independent Factor	SSI				
	Yes n (%)	No n (%)	P value	Odds Ratio	95% CI
	N=14	N=114			
Age					
>40	2 (14.3%)	24 (21.1%)	0.553		
40 or less	12 (85.7%)	90 (78.9%)		1.6	0.34-7.64
Gender					
M	3 (21.4%)	26 (22.8%)	0.907		
F	11 (78.6%)	88 (77.2%)		1.08	0.28-4.18

Pre-operative predictors of SSI

Antibiotic prophylaxis was used in 112 patients out of a total of 128. The SSI rate for the absence of prophylaxis was 6.2% while the SSI rate for two or more antibiotics use was 14% (Table 3). Despite no statistical significance, there were increased odds of developing SSI from antibiotic prophylaxis or the use of more than one type of antibiotics: odds ratios of 1.97 and 1.77, respectively. In addition, SSI rates for the ASA score of I and II were 9.3% and 12%, respectively. There was only one patient with an ASA score of III in the entire study population, who then didn’t develop SSI (Table 3).

**Table 3:** Association of pre-operative factors with Surgical Site Infection at Shirati KMT Hospital n: number of patients in percentage; N: number of patients with or without SSI; Abx: antibiotic prophylaxis; #, number; ASA: American Society of Anesthesiologists score; *, ASA score of II or III; CI: confidence interval

Variable	SSI		P value	Odds Ratio	95% CI
	Yes n (%)	No n (%)			
	N=14	N=114			
Abx					
Yes	13 (92.9%)	99 (86.8%)	0.521	1.97	0.24-16.2
No	1 (7.1%)	15 (13.2%)			
# of Abx					
2 or more	8 (57.1%)	49 (43.0%)	0.314	1.77	0.58-5.43
<2	6 (42.9%)	65 (57.0%)			
ASA score					
I	4 (28.6%)	39 (34.2%)	0.673		
>II*	10 (71.4%)	75 (65.8%)		1.30	0.38-4.31

Intra-operative predictors of SSI

In contrast to emergency and shorter surgeries of less than 30 minutes, there were increased odds of developing SSI with elective cases (odds ratio of 1.71) or surgeries lasting longer than 30 minutes (odds ratio of 1.24), with SSI rates of 13.8% and 12%, respectively (Table 4). While the general anesthesia (GA) SSI rate was 12%, surgeries requiring spinal (SA) and or local anesthesia (LA) had an SSI rate of 10.3. Compared to patients with clean wound classification, patients with clean contaminated wounds were 6.17 times more likely to develop SSI (p=0.034). SSI rates for clean and clean-contaminated wounds were 9.8% and 40%, respectively.

**Table 4:** Association of intra-operative factors with Surgical Site Infection at Shirati KMT Hospital n: number of patients in percentage; N: number of patients with or without SSI; CI: confidence interval; GA: general anesthesia; SA: spinal anesthesia; LA: local anesthesia

Variable	SSI		P value	Odds Ratio	95% CI
	Yes n (%)	No n (%)			
	N=14	N=114			
Operation Type					
Emergency	6 (42.9%)	64 (56.1%)	0.346		
Elective	8 (57.1%)	50 (43.9%)		1.71	0.56-5.24
Surgery Duration					
>30	8 (57.1%)	59 (51.8%)	0.703	1.24	0.41-3.81
<30	6 (42.9%)	55 (48.2%)			
Wound Class					
Clean	12 (85.7%)	111 (97.4%)	0.034		
Clean Contaminated	2 (14.3%)	3 (2.6%)		6.17	0.94-40.6
Anesthesia Type					
GA	6 (42.9%)	44 (38.6%)	0.758	1.19	0.38-3.79
SA & LA	8 (57.1%)	70 ( 61.4%)			

Multivariate logistic regression

On multivariate logistic regression, antimicrobial prophylaxis, major surgeries, and elective cases were 1.05, 1.16, and 1.07 times more likely to result in SSI (Table 5). Clean contaminated wound class is a significant predictor of SSI, with an odds ratio of 1.35 and p-value of 0.037.

**Table 5:** Multivariate logistic regression analysis of factors associated with Surgical Site Infection at Shirati KMT Hospital CI: confidence interval

Predictor variable	p-value	Odds Ratio	95% CI	
			Lower 95%	Upper 95%
Antibiotic prophylaxis	0.545	1.05	0.89	1.25
Major operation	0.093	1.16	0.98	1.37
Elective operation	0.236	1.07	0.96	1.20
Clean contaminated wound	0.037	1.35	1.02	1.78

## Discussion

Despite major improvements in clinical settings, SSI is one of the leading causes of nosocomial infections across the globe [18]. SSI has been reported to be higher and increasingly associated with morbidity and mortality in developing nations. Several studies in Tanzania previously reported SSI rates of 19.4%, 24% [14,15], and most recently 26% [16]. The purpose of this study was to establish a baseline SSI rate and to report on predictor variables associated with SSI among patients undergoing both major and minor surgeries at Shirati KMT Hospital in Northeastern Tanzania.

There was a total of 428 surgeries that occurred between January 1 and June 9, 2019, at Shirati KMT Hospital. After transcribing the handwritten hospital records into an Excel sheet and excluding missing information, we had a total of 128 patients (Table 1). SSI was documented in 14 of them, giving the SSI rate of 10.9%, which is the lowest when compared to previously reported SSI rates in the sub-Saharan region: 15.6-26% [14-16,19-21]. Although there was no statistically significant correlation between SSI and age or gender, the results showed that most of the SSI population was female (78.6%) with an SSI rate of 11.1%. The mean age of the SSI population was 28.6 with a standard deviation of 17.4 (Table 1). A recent study at Bugando Medical Centre in Northwestern Tanzania showed that their SSI group had a mean age of 38 and comprised 60% males [16] while another study from Ethiopia reported mean age of 30.85 and 61% males in their SSI population [21]. These contradictory findings can be due to the great abundance of certain types of surgeries, some of which are only appropriate for certain age or gender groups. For example, the majority (30.8%) of the patients with SSI had undergone prostatectomy at the Bugando Medical Centre in Northwestern Tanzania [16]. Whereas, in the current study, most of the SSI population are females of child-bearing ages and thus could have undergone cesarean section since such an operation has been reported to be associated with an increased risk of SSI [8].

Patients receiving antibiotic prophylaxis were at an increased odds of developing SSI (odds ratio of 1.97), consistent with previous studies in developing nations such as Tanzania, Ethiopia, and Nepal [12,15,20]. We also report an increased correlation between SSI and the use of two or more types of antibiotics. A higher rate of SSI among those with prophylaxis and the use of multiple antibiotics is possibly due to antibiotic resistance and multidrug-resistant bacteria, as previously reported [16,22]. There was a positive association between an ASA score above I and SSI, as shown by an odds ratio of 1.3 (Table 3), consistent with previous findings in both developed and developing nations that showed an increased risk of developing SSI in patients with pre-existing conditions such as obesity, diabetes mellitus, hypertension, and acquired immunodeficiency syndrome (AIDS) [16,23,24].

As previously shown in other studies that increased length of operation was associated with increased risk of SSI [16,21,25], we also found that patients undergoing operations of 30 minutes or longer were more prone to develop SSI (odds ratio of 1.24). This can be explained by major cases requiring extensive work in the operation room. In addition, patients who had undergone elective operations or received general anesthesia were at more risk of developing SSI compared to those undergoing spinal or local anesthesia. This contradicts the abovementioned assumption that elective operations such as cesarean section requiring spinal anesthesia can play a major role in the current findings. Although it is unclear whether general anesthesia can increase the risk of SSI, studies have shown that certain general anesthetics can alter neutrophil functions and phagocytosis, attenuating immunity [26]. Based on previous studies and our current findings that show an increased correlation between SSI and antibiotic prophylaxis, major operation, elective cases, or clean contaminated wound status, we carried out a multivariate logistic regression analysis. Among the possible predictors of SSI, clean contaminated wound class was significantly associated with SSI, as shown by both univariate and multivariate logistic regression analyses (Tables 4, 5). This finding was consistent across many reports in both developed and developing countries and is proven to be a major predictor for postoperative infection [15,16,27].

The present study was limited in that there was missing information on many patients undergoing surgeries at the Shirati KMT Hospital in the given period. We speculate that handwritten charts and notes play a major role in such limitations. As a result, misinterpretation of such data was highly possible and could have hindered our findings. To avoid such errors, we only included patients with complete information pertaining to the predictive factors. In addition, the misplacement of files was common. Files of deceased patients were dumped in one huge box without any organization so were not located due to time constraints. This could have been an important factor since mortality was found to be associated with SSI. Another crucial factor that added to the limitation of the current study was the absence of post-operation follow-ups and record-keeping of such information. SSI typically occurs within 30 days after surgery [1]. Most patients at the Shirati KMT Hospital did not stay in the post-op ward for this long, possibly due to a lack of hospital resources and financial limitations of the patients. The patients could have developed SSI and did not address it with the hospital or might have gone to a different hospital for treatment. Therefore, this study did not include analysis of previously reported and widely known SSI factors such as pre-morbid illnesses, cigarette smoking, prevalent of AIDS, pre-operative hospitalization details, number of people in the operation room, and use of povidone or surgical drain [16,24,28-30].

## Conclusions

Although there have been significant efforts in improving surgical practices around the world, SSI remains a major burden in resource-limited regions, particularly in Sub-Saharan countries such as Tanzania. In this retrospective analysis of surgeries occurring between January 1 and June 9, 2019, at Shirati KMT Hospital in Northeastern Tanzania, we conclude that clean contaminated wound class was a significant risk factor for SSI. Other factors that showed trends toward SSI were an age of 40 or younger, elective operations, operations lasting longer than 30 minutes, and use of antibiotic prophylaxis. This study is the first to determine the SSI rate and risk factors associated with SSI at the Shirati KMT Hospital and can be a crucial step for the hospital to improve its record-keeping and surveillance system for SSI. As such, an additional study exploring more widespread risk factors and patients' past medical history, social history including smoking and illicit drug use, and detailed hospitalization records is needed for developing and/or improving the surveillance system for SSI at the Shirati KMT Hospital.
